# Nicotinic Acetylcholine Receptor (nAChR) Dependent Chorda Tympani Taste Nerve Responses to Nicotine, Ethanol and Acetylcholine

**DOI:** 10.1371/journal.pone.0127936

**Published:** 2015-06-03

**Authors:** Zuo Jun Ren, Shobha Mummalaneni, Jie Qian, Clive M. Baumgarten, John A. DeSimone, Vijay Lyall

**Affiliations:** Department of Physiology and Biophysics, Virginia Commonwealth University, Richmond, VA, 23219, United States of America; Universidade do Estado do Rio de Janeiro, BRAZIL

## Abstract

Nicotine elicits bitter taste by activating TRPM5-dependent and TRPM5-independent but neuronal nAChR-dependent pathways. The nAChRs represent common targets at which acetylcholine, nicotine and ethanol functionally interact in the central nervous system. Here, we investigated if the nAChRs also represent a common pathway through which the bitter taste of nicotine, ethanol and acetylcholine is transduced. To this end, chorda tympani (CT) taste nerve responses were monitored in rats, wild-type mice and TRPM5 knockout (KO) mice following lingual stimulation with nicotine free base, ethanol, and acetylcholine, in the absence and presence of nAChR agonists and antagonists. The nAChR modulators: mecamylamine, dihydro-β-erythroidine, and CP-601932 (a partial agonist of the α3β4* nAChR), inhibited CT responses to nicotine, ethanol, and acetylcholine. CT responses to nicotine and ethanol were also inhibited by topical lingual application of 8-chlorophenylthio (CPT)-cAMP and loading taste cells with [Ca^2+^]_i_ by topical lingual application of ionomycin + CaCl_2_. In contrast, CT responses to nicotine were enhanced when TRC [Ca^2+^]_i_ was reduced by topical lingual application of BAPTA-AM. In patch-clamp experiments, only a subset of isolated rat fungiform taste cells exposed to nicotine responded with an increase in mecamylamine-sensitive inward currents. We conclude that nAChRs expressed in a subset of taste cells serve as common receptors for the detection of the TRPM5-independent bitter taste of nicotine, acetylcholine and ethanol.

## Introduction

Alcohol and nicotine are often co-abused. About 80% of alcohol-dependent people are also smokers and smokers have increased risk of developing alcohol use disorders [1–4]. Neuronal nAChRs, the molecular targets of nicotine that initiate dependence in smokers, may also contribute to the abusive properties of alcohol. Mecamylamine (Mec) blocks ethanol-induced dopamine release from the nucleus accumbens [5, 6]. Mec, CP-601932, sazetidine A and varenicline reduce alcohol and nicotine consumption and seeking [7–10] and the pleasurable effects of alcoholic beverages in patients [11]. Thus, nAChRs represent common targets at which ethanol and nicotine functionally interact in the CNS [8, 12] and in TRCs [13–15] and provide an important molecular link between the bitter taste of nicotine and alcohol and their systemic effects of addiction, co-dependence and relapse. In contrast, the olfactory responses to nicotine are independent of nAChRs [16]. Chronic nicotine upregulates midbrain nAChRs which may lead to increased dopaminergic neuron activation by ethanol [17]. The co-morbidity between alcohol and nicotine dependence can be attributed, in part, to common genetic factors [18].

Nicotine elicits bitter taste by activating two parallel bitter taste receptor-mediated pathways. One pathway is Transient Receptor Potential Cation Channel Subfamily M member 5 (TRPM5)-dependent and common to many other bitter tastants. The second pathway is TRPM5-independent, and depends upon the presence of nicotinic acetylcholine receptors (nAChRs) expressed in a subset of taste receptor cells (TRCs) [15]. It is also suggested that peripheral nAChRs may also regulate ethanol intake [19]. Thus, nAChRs represent common targets at which acetylcholine, nicotine and ethanol functionally interact in the central nervous system [20] and in the periphery.

Therefore, we investigated further if the nAChR-dependent pathway might be a common pathway involved in taste responses to nicotine, ethanol and acetylcholine. To this end, we monitored chorda tympani (CT) taste nerve responses to lingual stimulation with nicotine free base, ethanol, and acetylcholine in Sprague Dawley rats in the absence and presence of nAChR agonists and antagonists. To determine if these responses were independent of the classical bitter taste transduction pathway involving the T2R (G protein-coupled bitter taste receptors)-α-gustducin-PLCβ2 (phospholipase C, β2)-TRPM5 pathway, CT responses were also monitored in wild-type (WT) and TRPM5 knockout (KO) mice. The results presented here show that CT responses to nicotine, ethanol, and acetylcholine are blocked by the nAChR modulators: mecamylamine (Mec), dihydro-β-erythroidine (DHβE), and CP-601932 (a partial agonist of α3β4* nAChR) [7]. CT responses to nicotine and ethanol were inhibited by topical lingual application of 8-chlorophenylthio (CPT)-cAMP and loading TRCs with Ca^2+^ by topical lingual application of ionomycin + CaCl_2_. In contrast, CT responses to nicotine were enhanced when TRC [Ca^2+^]_i_ was reduced by the topical lingual application of BAPTA-AM. We conclude that TRPM5-independent neural responses to nicotine, acetylcholine and ethanol are partially dependent upon the presence of nAChR subunits in a subset of fungiform taste bud cells.

## Materials and Methods

### Chemicals

(-) Nicotine free base (NFB) ((−)-1-Methyl-2-(3-pyridyl)pyrrolidine, (S)-3-(1-Methyl-2-pyrrolidinyl)pyridine), ethanol, acetylcholine, 8-(4-Chlorophenylthio) adenosine 3',5'-cyclic monophosphate (8-CPT-cAMP), 1,2- Bis(2- aminophenoxy) ethane- N,N,N′,N′-tetraacetic acid tetrakis-(acetoxymethyl ester) (BAPTA-AM), ionomycin, H89 (*N*-[2-[[3-(4-Bromophenyl)-2-propenyl]amino]ethyl]-5-isoquinolinesulfonamide dihydrochloride), mecamylamine (Mec), dihydro-β-erythroidine (DHβE), HEPES (4-(2-hydroxyethyl)-1-piperazineethanesulfonic acid), and CellTak were obtained from Sigma-Aldrich. CP-601932, a partial agonists of α3β4* nAChR, was obtained from Pfizer Inc. (* indicates the possibility of additional subunits).

### Animals

The forty female Sprague-Dawley rats (150–200 g) used in this study were obtained from Charles River Laboratories, Wilmington, MA, USA. C57BL/6 mice homozygous for a partial deletion of the TRPM5 gene (KO) were bred from animals generously provided by C.S. Zuker (Columbia University, New York). These KO mice have an internal deletion of the TRPM5 gene, lacking exons 15–19 encoding transmembrane segments 1–5 [21]. Similar to the case with PLCβ2 KO mice, these TRPM5 KO mice lack behavioral and peripheral neural responses to quinine and other prototypical bitter tastants [15, 21]. The control wild-type (WT) mice were C57BL/6J and were obtained from Charles River Laboratories. Six male and female WT and KO mice were used for CT experiments. Rats and mice were maintained on a 12 h light/dark schedule and were given a pellet diet and water *ad libitum*.

### CT taste nerve recordings

Animals were housed in the Virginia Commonwealth University (VCU) animal facility in accordance with institutional guidelines. All *in vivo* and *in vitro* animal protocols were approved by the Institutional Animal Care and Use Committee (IACUC) of VCU. For CT recordings, rats were anesthetized by intraperitoneal injection of sodium pentobarbital (60 mg/kg), and supplemental sodium pentobarbital (20 mg/kg) was administered as necessary to maintain surgical anesthesia. The animal’s corneal reflex and toe pinch reflex were used to monitor the depth of surgical anesthesia. Body temperatures were maintained at 37° with a Deltaphase isothermal pad (model 39 DP; Braintree Scientific, Braintree, MA). The left CT nerve was exposed laterally as it exited the tympanic bulla and was placed onto a 32-gauge platinum-iridium wire electrode. An indifferent-electrode was placed in nearby tissue. Neural responses were differentially amplified with an optically coupled isolated bioamplifier (ISO-80; World Precision Instruments, Sarasota, FL). Stimulus solutions were injected into a Lucite chamber (3 ml; 1 ml/s) affixed by vacuum to a 30-mm^2^ patch of anterior dorsal lingual surface. CT responses were recorded under zero lingual current-clamp and analyzed as described previously [22, 23].

CT responses were also monitored in WT and TRPM5 KO mice. Mice (30–40 gm) were anesthetized by intraperitoneal injection of pentobarbital (30 mg/Kg) and supplemental pentobarbital (10 mg/Kg) was administered as necessary to maintain surgical anesthesia. Our lingual perfusion chamber is too big for the mouse tongue. Therefore, CT recordings were made in mice while either rinse solutions or solutions containing taste stimuli were flowed over the anterior tongue at a rate of 1 ml/s using a syringe filled with 3 ml of solution. The rest of the procedure was the same as in rats. At the end of each experiment animals were humanely killed by an intraperitoneal overdose of pentobarbital (approximately 195 mg/Kg body weight for rats and 150 mg/Kg weight for mice).

Integrated responses were typically recorded for 1–2 min and were quantified by calculating the mean over the final 30 s of the response. Mean responses were then normalized by dividing them by the mean response to 300 mM NH_4_Cl over a similar final 30 s period. The normalized data were reported as the mean ± standard error of the mean (SEM) of the number of animals. Responses to control stimuli consisting of 300 mM NH_4_Cl applied at the beginning and at the end of the experiment were used to assess preparation stability. The preparation was considered stable only if the difference between the magnitude of the control stimuli at the beginning and at the end of the experiment was less than 10% [22, 23]. Integrated neural responses and lingual current and voltage changes were captured on disk using LabView software (National Instruments, Austin, TX) and analyzed off-line as described previously [22, 23].

The rinse solution was H_2_O, and stimulating solutions contained nicotine free base (NFB; 10 or 20 mM), ethanol (20–60%) or acetylcholine (ACh; 1–5 mM). CT responses were monitored in the presence of Mec (0–0.5 mM), DHβE (0–0.5 mM), and CP-601932 (0–0.01 mM). CT responses to nicotine and alcohol were also monitored after topical lingual application of 15 mM 8-CPT-cAMP for 30 min [24], 33 mM BAPTA-AM for 30 min or 150 μM ionomycin +10 mM CaCl_2_ for 30 min [22–24]. In some rats we monitored CT responses to nicotine and alcohol before and following 2 h post-subcutaneous injection of saline or CP-601932 (10 mg/Kg body weight in saline) [7].

### Data Analysis

The magnitude of the normalized tonic CT responses to nicotine, alcohol, and acetylcholine was expressed as the mean ± SEM of the number of animals in each group (*N*) and Student’s ‘t’ test was used to obtain the statistical significance.

### Patch-clamp studies on isolated rat fungiform TRCs

Four rats were anesthetized by exposing them to an inhalation anesthetic, isoflurane (1.5 ml) in a desiccator. When the rats were fully unconscious, a midline incision was made in the chest wall and the aorta severed. The tongues were then rapidly removed and stored in ice-cold control Ringer’s solution containing (in mM): 140 NaCl, 5 KCl, 1 CaCl_2_, 1 MgCl_2_, 10 sodium pyruvate, 10 glucose, 10 HEPES, pH 7.4. The anterior lingual epithelium was isolated by collagenase treatment. Taste buds were harvested from rat fungiform papillae, aspirated with a micropipette and individually transferred onto coverslips, avoiding contaminating cells and debris as described earlier [25]. Taste bud fragments and single TRCs were transferred to a glass cover slip coated with cellTak that formed the base of a perfusion chamber and superfused at ~2 ml/min (21–22°C). Whole cell patch-clamp recordings were performed as described earlier [26]. Patch electrodes were made from thin-walled 7740 borosilicate glass (Sutter) and fire polished and had an initial resistance of 2–3 MΩ. Whole cell currents recorded with an Axoclamp 200B amplifier (Axon) were low pass filtered at 2 kHz (Bessel) and digitized at 5 kHz. Voltage-clamp protocols and data acquisition were governed by a Digidata 1321A digitizer and pCLAMP 8.0 software (Axon) [27, 28]. Isolated TRCs were bathed in Tyrode solution containing: 140 mM NaCl, 5 mM KCl, 10 mM Na-pyruvate, 2 mM MgCl_2_, 10 mM HEPES, 10 mM glucose, 1 mM CaCl_2_, pH 7.4. The pipette solution contained: 140 mM KCl, 11 mM EGTA, 10 mM HEPES, 5 mM MgATP, 2 mM MgCl_2_, 1 mM CaCl_2_, pH 7.2.

## Results

### CT responses to nicotine involve both TRPM5-dependent and TRPM5-independent bitter taste transduction mechanisms

#### Studies in WT and TRPM5 KO mice

**Fig 1:**
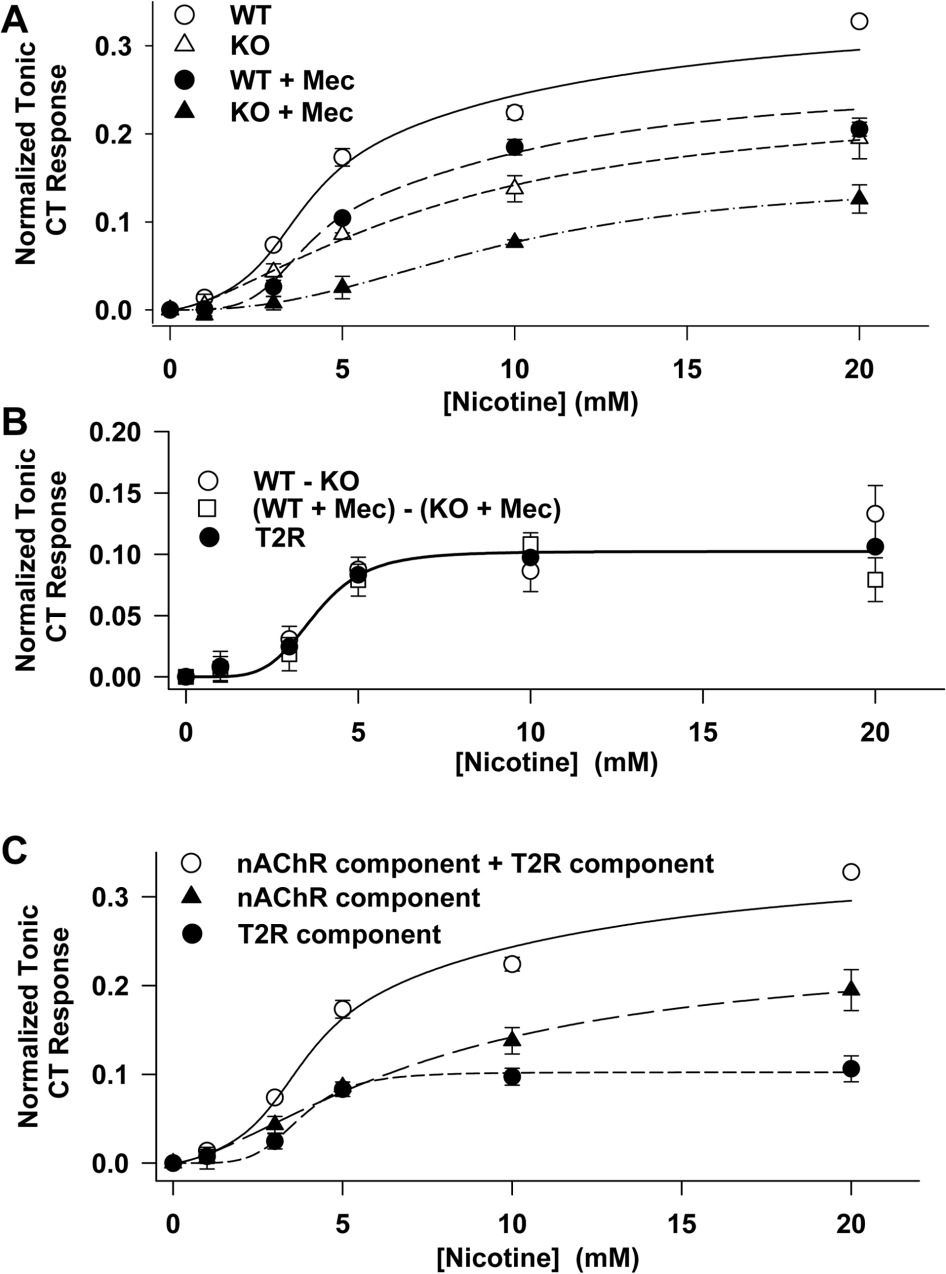
TRPM5-dependent and TRPM5-independent components of the nicotine tonic CT response in mice. **(A)** Tonic CT response to lingual stimulation with increasing nicotine concentration in WT mice (○) and TRPM5 KO mice (△). Tonic CT response to stimulus solutions containing increasing nicotine concentration containing, in addition, 0.3 mM Mec in WT mice (●) and TRPM5 KO mice (▲). Curves through the data points in the absence and presence of Mec in TRPM5 KO mice are nonlinear regression fits to Eq 1 with parameters listed in Table 1 (see text). In the WT mice, the curve through the data points is the sum of the curves through the points in TRPM5 KO mice and the T2R points (see Fig 1B). The curve through the data points in the WT mice in the presence of Mec is the sum of the curves through the data points in TRPM5 KO mice in the presence of Mec and the T2R points. **(B)** The difference in CT responses between WT and KO mice with increasing nicotine concentration (○; WT—KO, mean ± SE, *N* = 3) and the difference in CT responses between WT and KO mice with increasing nicotine concentration containing, in addition, 0.3 mM Mec (□; (WT + Mec—TRPM5 KO + Mec, mean ± SE, *N* = 3) and the mean T2R response (●; Average T2R, i.e. (((WT—KO) + (WT + Mec—TRPM5 KO + Mec))/2 ± SE). Applying a *t*-test at each nicotine concentration (1, 3, 5, 10, and 20 mM) shows the mean WT—KO difference is not significantly different from the mean ((WT + Mec)—TRPM5 KO + Mec)) difference at all nicotine concentrations. A two-factor ANOVA on the means shows that both (WT—KO) and ((WT + Mec)—TRPM5 KO + Mec)) vary significantly with nicotine concentration (P = 0.0052) but this concentration dependence is not significantly different between conditions with and without Mec (P = 0.414). The *t*-test and ANOVA each justify the representation of the T2R contribution to the response as the mean: ((WT—KO) + (WT + Mec)—TRPM5 KO + Mec))/2 ± SE plotted here. The curve is the nonlinear regression fit of the mean to Eq 1 using the parameters for T2R in Table 1. **(C)** The CT response in WT mice with increasing nicotine concentration (○) represents the sum of the output from nAChR taste receptors (TRPM5-independent component) and T2R receptors (TRPM5-dependent component). The response in TRPM5 KO mice (▲) represents only nAChR-dependent response, and the mean T2R receptor contribution to the CT response as described in Fig 1B. The curve through the points representing the nAChRs is the nonlinear regression fit to Eq 1 using the parameter values for nAChR in the absence of Mec in Table 1. The curve through the mean T2R points (●) is the nonlinear regression fit to Eq 1 using the parameter values for T2R in Table 1. The curve through the WT data points (○) is the sum of the curves for nAChRs-dependent and T2R-dependent CT responses.

Consistent with our previous studies [15], stimulating the tongue with increasing nicotine concentrations elicited dose-dependent increases in CT responses recorded in WT and TRPM5 KO mice (Fig 1A). At the maximum concentration of nicotine used, the mean tonic CT was 40% lower in TRPM5 KO mice compared with WT mice. The nicotine CT response in both WT and TRPM5 KO mice was significantly inhibited by Mec. This suggests that the TRPM5-independent taste responses to nicotine are derived from its interactions with nAChRs in TRCs [13–15]. Mec is expected to inhibit specifically the same subset of nAChR taste receptors in WT and TRPM5 KO mice. Therefore, the difference between the CT response to nicotine in Fig 1A between WT and TRPM5 KO mice should represent only the contribution of the T2Rs to the total CT response. Fig 1B shows that the difference in the CT response to nicotine between WT and TRPM5 KO mice in the absence of Mec (WT- KO) is not significantly different from the difference in the nicotine CT responses in the presence of Mec ((WT + Mec)-(KO + Mec)). These results suggest that WT and TRPM5 KO mice share the same nAChR distribution, and that this difference represents the nicotine CT response due to its interactions with T2Rs. Fig 1C shows that in WT mice, the CT response to nicotine represents the output of both T2R and nAChR bitter taste receptors. The response in TRPM5 KO mice represents the nAChR component alone, and the T2R component is the mean of the response (WT-KO) difference and the ((WT + Mec)-(KO + Mec)) difference.

In Fig 1A, 1B and 1C the CT response functions show positive cooperativity as the nicotine concentration increases above zero and a tendency toward saturation with increasing nicotine concentration. These properties can be represented quantitatively for each receptor class by the Hill equation as follows:

$$r_{i}=\frac{b_{i}\;c^{n}}{k_{i}{}^{n}+c^{n}}$$ (1)

Here *i* has either the value *t* for the T2R component or *a* for the nAChR component. *r*_*i*_ is the CT response to receptor class *i* at nicotine concentration, *c*, *b*_*i*_ is the maximum response of the receptor class *i*, *k*_*i*_ is the dissociation constant between the receptor class *i* and nicotine, and *n* values greater than one indicate positive cooperativity among receptor subunits. Table 1 gives the nonlinear regression fit parameter values for each receptor type in the absence and presence of Mec in the nicotine stimulus solutions.

**Table 1:** Least squares fit parameter values for TRPM5-dependent and TRPM5-independent components of the nicotine CT response.

Receptor type (Stimulus)	*b*_*i*_	*k*_*i*_ (mM)	*n*_*i*_	*b*_*i*_/(*b*_*t*_ + *b*_*a*_)	R^2^
nAChR (*a*) (Nic)	0.239 ± 0.021	7.81 ± 1.16	1.5 ± 0.2	0.70 ± 0.08	0.99
nAChR (*a*) (Nico + Mec)	0.147 ± 0.011	9.62 ± 0.88	2.4 ± 0.3	0.59 ± 0.05	0.99
T2R (*t*) (Nic)	0.102 ± 0.004	3.76 ± 0.19	5.0 ± 0.9	0.30 ± 0.02	0.99
T2R (*t*) (Nic + Mec)				0.41 ± 0.02	

*i* = TRPM5-dependent (*t*) or TRPM5-independent component (*a*)

*r*_*i*_ = CT response to receptor class *i* at nicotine concentration, *c*

*b*_*i*_ = maximum response of the receptor class *i*

*k*_*i*_ = dissociation constant between the receptor class *i* and nicotine

*n* >1 = positive cooperativity among receptor subunits

(b_*t*_ + b_*a*_) = (maximum TRPM5-dependent component + maximum TRPM5-independent component)

Nic = nicotine; Mec = mecamylamine

At nicotine concentrations less than 5 mM, nAChRs and T2Rs each account for about 50% of the total response to nicotine. Above 5 mM nicotine the T2R fraction has reached its maximum response and the nAChRs begin to predominate. Consequently at 10 mM nicotine the T2R response fraction has fallen to 41% and at 20 mM nicotine it is 35% and 30% in the high concentration limit (Table 1). Since the TRPM5-dependent response (T2R component) is Mec-insensitive (Fig 1B), in the presence of Mec, the T2R component in the high concentration limit represents a higher fraction of the CT response, i.e. 41%.

### CT responses to nicotine in TRPM5 KO mice are blocked by DHβE and Mec

**Fig 2:**
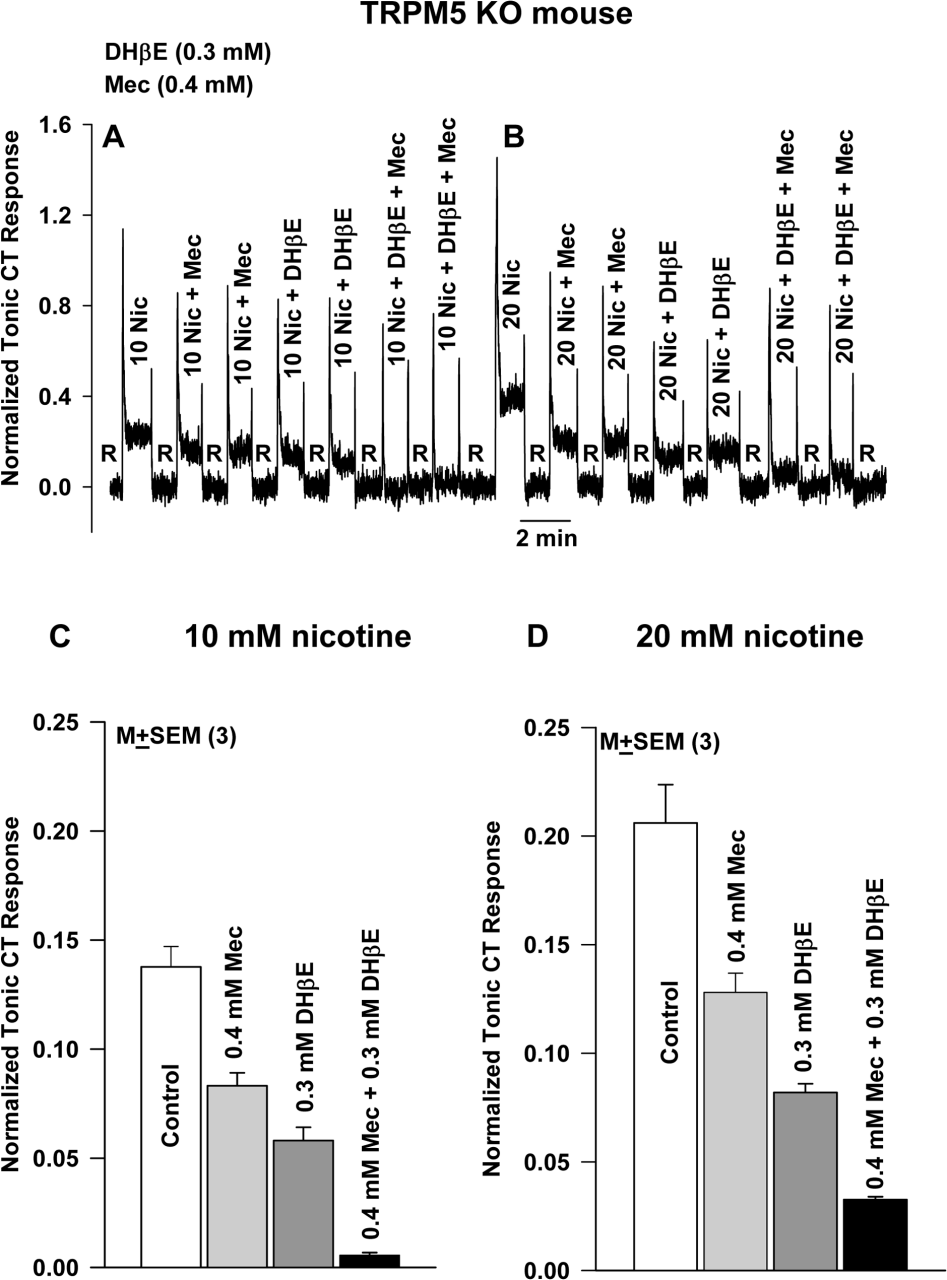
Effect of DHβE and Mec on the CT response to nicotine in TRPM5 KO mice. This shows a representative normalized CT response in a TRPM5 KO mouse in which the tongue was first rinsed with distilled H_2_O (R) and then stimulated with 10 mM **(A)** or 20 mM **(B)** nicotine in the absence and the presence of 0.4 mM Mec or 0.3 mM DHβE or 0.4 mM Mec + 0.3 mM DHβE. Also shown are the mean normalized CT response to 10 mM **(C)** and 20 mM **(D)** nicotine in the absence and presence of Mec alone, DHβE alone, and Mec + DHβE in TRPM5 KO mice (*N* = 3). Relative to 10 mM nicotine **(C)**, the p values for the mean normalized CT responses in the presence Mec, DHβE, and Mec + DHβE were 0.008, 0.002, and 0.002, respectively. Relative to Mec + DHβE, the p values for the mean normalized CT responses in the presence Mec or DHβE alone were 0.0002 and 0.0011, respectively (unpaired; *N* = 3). Relative to 20 mM nicotine **(D)**, the p values for the mean normalized CT responses in the presence Mec, DHβE and Mec + DHβE were 0.017, 0.002, and 0.006, respectively. Relative to Mec + DHβE, the p values for the mean normalized CT responses in the presence Mec or DHβE alone were 0.0016 and 0.0072, respectively (unpaired; *N* = 3).

Consistent with our previous studies [15], stimulating the TRPM5 KO mouse tongue with 10 mM (Fig 2A) and 20 mM nicotine (Fig 2B) produced a dose-dependent increase in the CT response. Adding 0.4 mM Mec (a non-specific nAChR antagonist) to 10 mM (Fig 2A) or 20 mM (Fig 2B) nicotine stimulation solutions inhibited CT responses to nicotine. The mean normalized tonic CT responses to nicotine in TRPM5 KO mice are shown in Fig 2C and 2D. Mec inhibited the CT response to 10 mM (Fig 2C) and 20 mM (Fig 2D) nicotine by 39.5% and 37.8%, respectively (*N* = 3). The addition of 0.3 mM DHβE, a competitive α4β2 nicotinic antagonist [29], to 10 mM (Fig 2A) or 20 mM (Fig 2B) nicotine stimulation solutions also inhibited the CT responses to nicotine. When both drugs were added together, the inhibition in the nicotine tonic CT response was greater than that observed in the presence of Mec or DHβE alone. The mean normalized tonic CT responses to 10 and 20 mM nicotine in TRPM5 KO mice under control conditions, in the presence of Mec alone, DHβE alone, and Mec + DHβE are also shown in Fig 2C and 2D (*N* = 3). DHβE alone inhibited the mean normalized tonic CT response to 10 mM and 20 mM nicotine by 57.8% and 60.2%, respectively. In the presence of 0.4 mM Mec + 0.3 mM DHβE, the tonic responses to 10 mM and 20 mM nicotine were inhibited by 96.0% and 84.1%, respectively. These results confirm our previous studies that TRPM5-independent CT responses to nicotine are blocked by nAChR antagonists [15], and further suggest that more than one type of nAChR expressed in fungiform TRCs contribute to the generation of the nicotine CT response. Taken together, the above data indicate that the TRPM5-independent component of the nicotine CT response depends on nAChRs expressed in a subset of fungiform TRCs.

#### Studies in Sprague-Dawley (SD) rats

**Fig 3:**
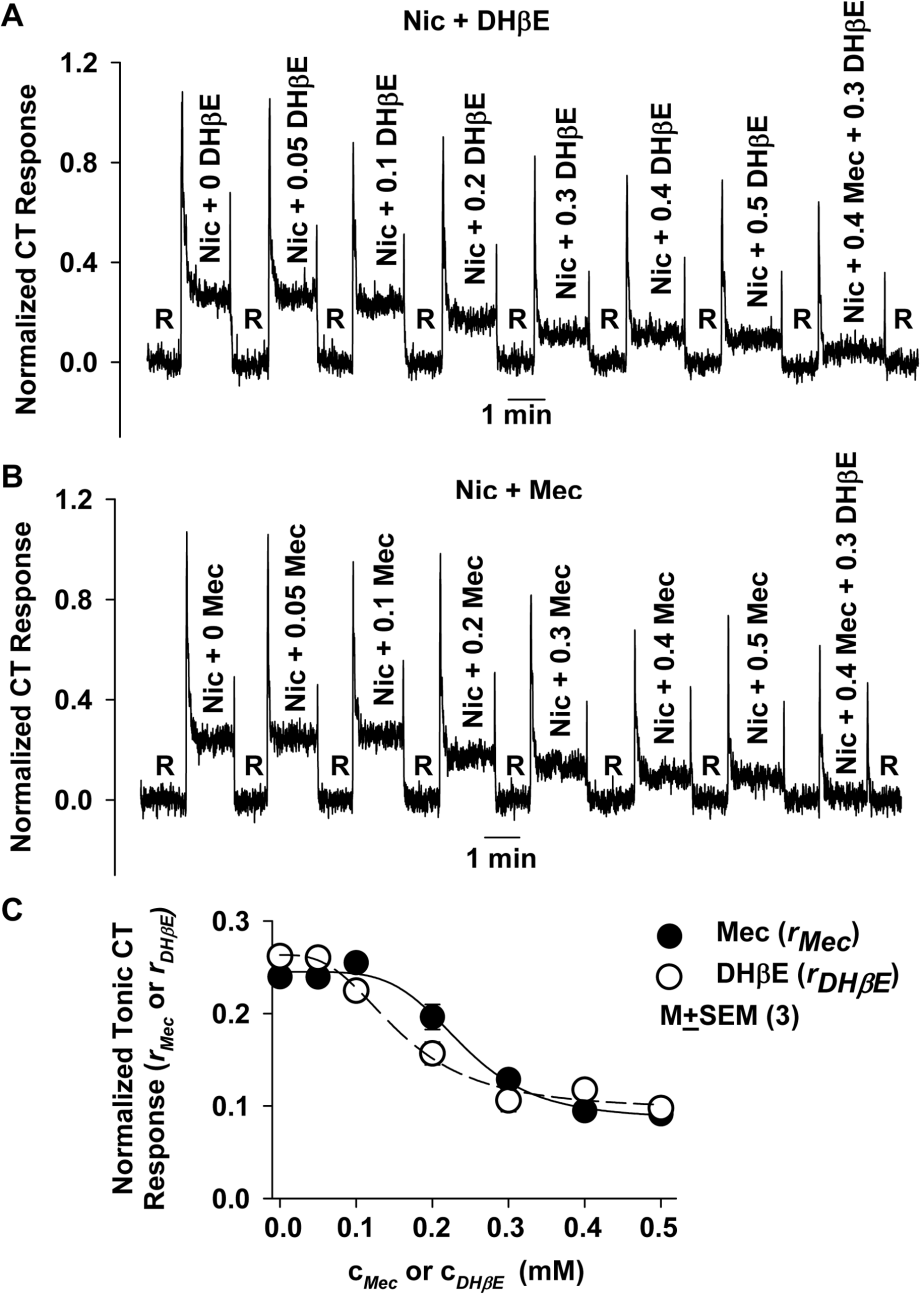
Effect of varying concentrations of DHβE or Mec on the CT response to nicotine in rats. **(A)** This is a representative normalized CT response in a rat in which the tongue was first rinsed with distilled H_2_O (R) and then stimulated with 10 mM nicotine solutions containing 0–0.5 mM DHβE or 0.3 mM DHβE + 0.4 mM Mec. **(B)** This is a representative normalized CT response in a rat in which the tongue was first rinsed with distilled H_2_O (R) and then stimulated with 10 mM nicotine solutions containing 0–0.5 mM Mec or 0.4 mM Mec + 0.3 mM DHβE. **(C)** Display of the mean normalized tonic CT responses (*N* = 3) to 10 mM nicotine in the presence of increasing concentrations (0–0.5 mM) of Mec (c_*Mec*_; ●) or DHβE (c_*DHβE*_; ○). The solid line is the least squares fit of Eq 2 to the Mec data set and the broken line is the fit to the DHβE data set. The least squares fit parameter values are shown in Table 2.

Experiments were performed in rats using a lingual perfusion chamber to reduce variability in the phasic component of the CT response and to increase reproducibility in the modulatory effects of nAChR antagonists and agonists. In our previous studies [15] Mec, a non-specific blocker of nAChRs, inhibited CT responses to nicotine. Here, we show that adding increasing concentrations of DHβE (0–0.5 mM), a competitive α4β2 nicotinic antagonist [29], in a 10 mM nicotine solution partially inhibited the nicotine CT response in a dose-dependent manner (Fig 3A). Even at the highest concentration of DHβE tested (0.5 mM), CT responses to nicotine were higher than those observed with water alone. This demonstrates that a component of the nicotine CT response is DHβE insensitive. The DHβE-insensitive response may depend on the T2R component and/or upon nAChRs that are insensitive to DHβE. Fig 3A further shows that stimulating the rat tongue with a 10 mM nicotine solution containing 0.3 mM DHβE + 0.4 mM Mec produced a further decrease in the nicotine CT response relative to 0.3 mM DHβE alone. These results show that the DHβE-insensitive component of the tonic nicotine CT response is, moreover, Mec-sensitive.

Mec also partially inhibited the nicotine CT response in a dose-dependent manner (Fig 3B). Even at the highest concentration of Mec tested (0.5 mM), a Mec-insensitive nicotine CT response was observed that was further inhibited in the presence of 0.4 mM Mec + 0.3 mM DHβE. These results show that the Mec-insensitive component of the tonic nicotine CT response is, as implied from the results of Fig 3A, DHβE-sensitive. Taken together, the above results indicate that similar to the case with WT mice, the CT response to nicotine in SD rats is derived from at least 3 classes of receptors, namely the TRPM5-dependent T2R bitter taste receptor, TRPM5-independent DHβE-sensitive nAChRs, and TRPM5-independent Mec-sensitive nAChRs expressed in a subset of fungiform taste bud cells.

Fig 3C shows the mean normalized tonic CT response for 10 mM nicotine as a function of the DHβE concentration (*c*_*DHβE*_) or the Mec concentration (*c*_*Mec*_). No change in the nicotine tonic CT response was observed at low antagonist concentration (≤ 0.05 mM DHβE and ≤ 0.1 mM Mec). In each case the data were fitted to a Hill-type equation expressing inhibitory kinetics (Eq 2) using least squares criteria:

$$r_{i}=r_{it}+\frac{a_{i}\;k_{i}{}^{n_{i}}}{k_{i}{}^{n_{i}}+c_{i}{}^{n_{i}}}$$ (2)

The subscript *i* refers to either the inhibitor Mec or DHβE, *r*_*i*_ is the response at a Mec or DHβE concentration *c*_*i*_, *a*_*i*_ is the maximum response sensitive to inhibitor *i*, which occurs at *c*_*i*_ = 0, *k*_*i*_ is the dissociation constant between the inhibitor *i* and an inhibitor *i*-sensitive nAChR, *n*_*i*_ is a constant >1 consistent with the positively cooperative nature of the inhibitor *i*-nAChR interaction. *r*_*it*_ is the sum *r*_*t*_ + *r*_*i0*,_ where *r*_*t*_ is the response due to the T2R-dependent component, which is likely insensitive to nAChR inhibitors, and *r*_*i0*_ is the part of the response due to those nAChRs that are either insensitive to Mec when Mec is the inhibitor applied to the tongue with nicotine or insensitive to DHβE when DHβE is the inhibitor applied to the tongue with nicotine. The dissociation constant between Mec and its receptor, *k*_*mec*_, was significantly larger than that for DHβE and its receptor, *k*_*DH*β*E*_ (see Table 2). This implies that DHβE on the average binds to its receptors with higher affinity than the affinity with which Mec binds its receptors.

**Table 2:** Fitting parameters for the 10 mM nicotine CT response in the presence of increasing concentrations of DHβE and Mec using Eq 2.

Inhibitor (*i*)	*r*_*it*_	*a*_*i*_	*k*_*i*_ (mM)	*n*_*i*_	*r*_*i0*_	*r*_*t*_	*r*_*i0*_/ (*r*_*i0*_ + *a*_*i*_)
Mec	0.086 ± 0.011	0.159 ± 0.013	0.240 ± 0.014	4.9 ± 1.2	0.059 ± 0.015	0.027 ± 0.010	0.272 ± 0.072
DHβE	0.096 ± 0.013	0.168 ± 0.017	0.157* ± 0.018	2.9 ± 0.8	0.069 ± 0.016	0.027 ± 0.010	0.293 ± 0.075

*i = Mec* or *DHβE*

*r*_*i*_ = response at Mec or DHβE concentration *c*_*i*_

*a*_*i*_ = maximum part of the response sensitive to inhibitor *i*, which occurs when *c*_*i*_ = 0

*k*_*i*_ = the dissociation constant between inhibitor *i* and an inhibitor-sensitive nAChR

*n*_*i*_ = a constant >1 consistent with the positively cooperative nature of the inhibitor-nAChR interaction

*r*_*it*_ = *r*_*t*_ + *r*_*i0*,_ where *r*_*t*_ the response due to T2Rs and *r*_*i0*_ is the part of the response insensitive to Mec when Mec is the inhibitor applied with nicotine or the part of the response insensitive to DHβE when DHβE is the inhibitor applied with nicotine

*p = 0.0216 (*N* = 3)

The fitting procedure yielded the *r*_*it*_ values in Table 2 and the response data for the mixture containing 10 mM nicotine + 0.4 mM Mec + 0.3 mM DHβE yielded *r*_*t*_, the value of the T2R component. The values for *r*_*i0*_, the part of the response due to the nAChRs insensitive to either Mec or DHβE, were found from the difference, *r*_*it*_—*r*_*t*_. From Table 2 the Mec-insensitive response fraction of the nAChR component (*r*_*Mec0*_ /(*r*_*Mec0*_ + *a*_*Mec*_)) was 0.272 ± 0.072. The DHβE-insensitive response fraction of the nAChR component was 0.293 ± 0.075. These fractions are not significantly different from each other. Given the standard error, we observe that the Mec-insensitive and the DHβE-insensitive components may be as small as about 20% or as large as about 33%, which suggests there may be three to five nAChRs involved in detecting nicotine as a bitter tastant.

We can estimate the T2R-dependent component as the nicotine CT response remaining in the presence of 0.3 mM DHβE + 0.4 mM Mec, i.e. the part of the response insensitive to both DHβE and Mec. This response was estimated to be 0.027 ± 0.010 (*N* = 4). This value is 11% of the response to 10 mM nicotine alone. These results suggest that nAChRs account for 89% of the nicotine CT response in SD rats. In contrast, in WT mice, the nAChR-dependent component at 10 mM nicotine accounted for 59% and the T2R component accounted for 41% of the nicotine CT response (Fig 1 and Table 1). It is important to note that although the mean tonic CT response to nicotine was of similar magnitude in SD rats (0.237 ± 0.015; *N* = 3; Fig 3) and WT mice (0.224 ±0.006; *N* = 3; Fig 1), the contribution of the T2R-bitter taste transduction mechanism to the tonic CT response in rats (11%) is much smaller than in WT mice (41%). These results show that the contribution of the T2R-dependent and the nAChR-dependent pathways to the CT response to nicotine may vary between species. In addition, within a species the respective contributions of these pathways vary with nicotine concentration due to the particular kinetics of agonist-receptor interaction (Fig 1 and Table 1).

### CT responses to acetylcholine are blocked by Mec and DHβE

**Fig 4:**
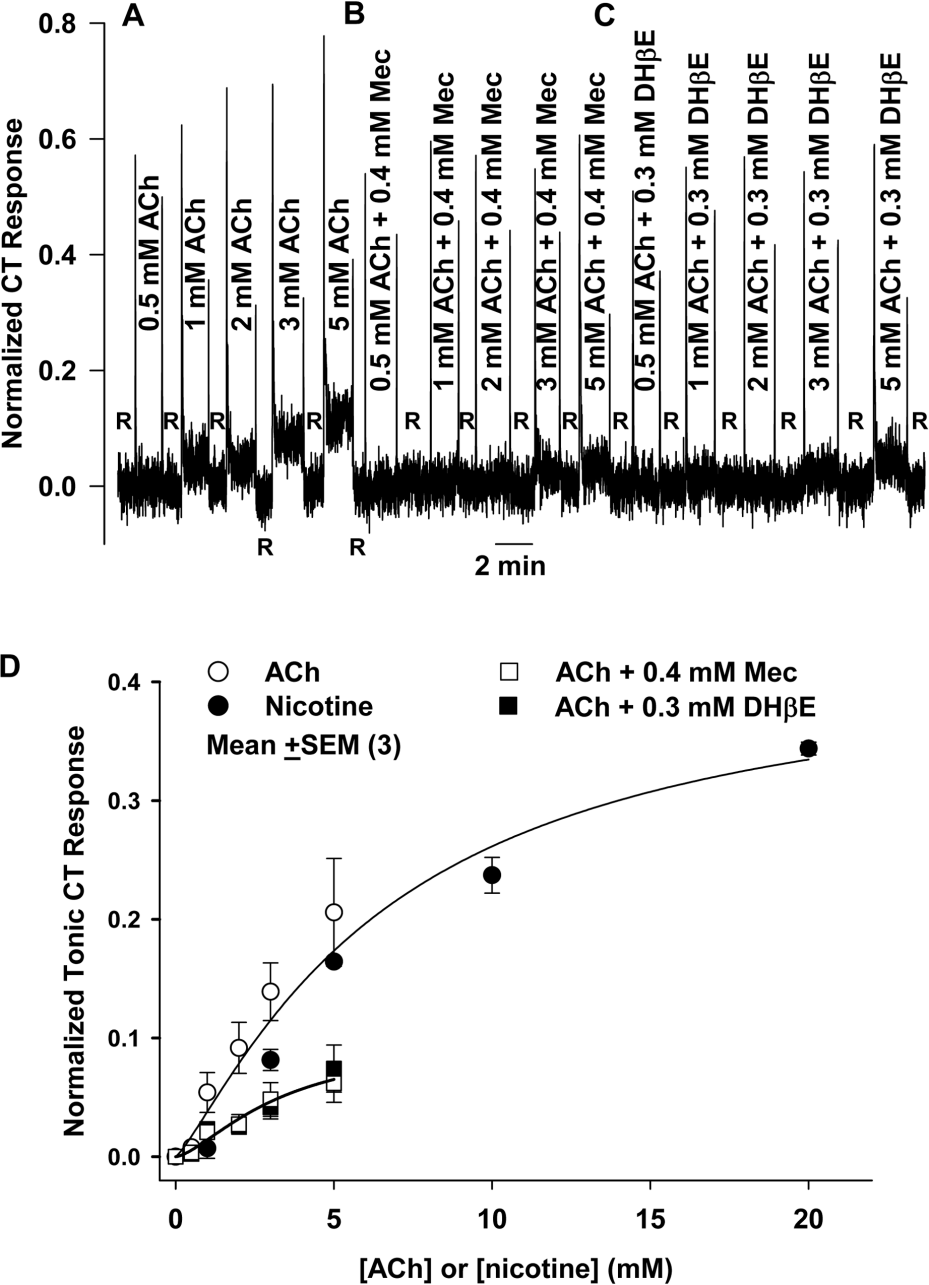
Effect of DHβE and Mec on the rat CT responses to acetylcholine (ACh). **(A)** Normalized CT responses to ACh in which the rat tongue was first rinsed with distilled H_2_O (R) and then stimulated with increasing concentrations of ACh (0.5–5 mM). Also shown are CT responses to ACh solutions (0.5–5 mM) containing either 0.4 mM Mec **(B)** or 0.3 mM DHβE **(C)**. Similar to responses to nicotine, responses to ACh are also partially inhibited by both Mec and DHβE. **(D)** Plots of mean normalized CT responses (*N* = 3) at varying ACh (○), nicotine (●), ACh + 0.4 mM Mec (□), and ACh + 0.3 mM DHβE (■). CT responses to ACh were not significantly different from responses to nicotine as a function of concentration. ACh CT responses in the presence 0.4 mM Mec or 0.3 mM DHβE were reduced to about 33% of control responses, similar to the case with nicotine.

Since nicotine elicited Mec- and DHβE-sensitive CT responses in WT mice and SD rats, we should expect that acetylcholine applied to the tongue as a taste stimulus will also elicit Mec- and DHβE-sensitive CT responses. Consistent with this, stimulating the rat tongue with increasing acetylcholine concentrations (0.5–5.0 mM) produced a dose-dependent increase in the CT response (Fig 4A). No response above baseline was observed at 0.5 mM acetylcholine, but between 0.5 mM and 1 mM acetylcholine the response rose rapidly and from 1 to 5 mM the CT responses continued to increase but with decreasing slope. This suggests that the CT response may saturate at still higher concentrations of acetylcholine than used in this experiment. The CT response to the same series of acetylcholine concentrations was significantly blocked by 0.4 mM Mec (Fig 4B) and 0.3 mM DHβE (Fig 4C). The CT response was inhibited to a similar extent by Mec and DHβE.

Fig 4D shows the concentration versus the tonic CT response curves for both nicotine (●) and acetylcholine (○). Between 1 and 5 mM nicotine or acetylcholine the tonic CT responses did not differ significantly. The least squares fit to the nicotine and acetylcholine data sets taken together was drawn using Eq 1 dropping the subscripts since in this case all quantities refer to the single joint nicotine and acetylcholine data set. The following parameters were found: *a* = 0.42, *k* = 7.7 mM, and *n* = 1.5. Similar to the case with nicotine-inhibitor mixtures, stimulus solutions containing either acetylcholine + Mec or acetylcholine + DHβE reduced the CT response to about 33% of the control, again consistent with the involvement of multiple nAChRs in responses to agonists. Moreover, these results suggest that both acetylcholine and nicotine interact with the nAChRs in fungiform taste bud cells and produce qualitatively and quantitatively similar CT responses in the absence and presence of Mec and DHβE.

### CT responses to ethanol are blocked by Mec and DHβE

**Fig 5:**
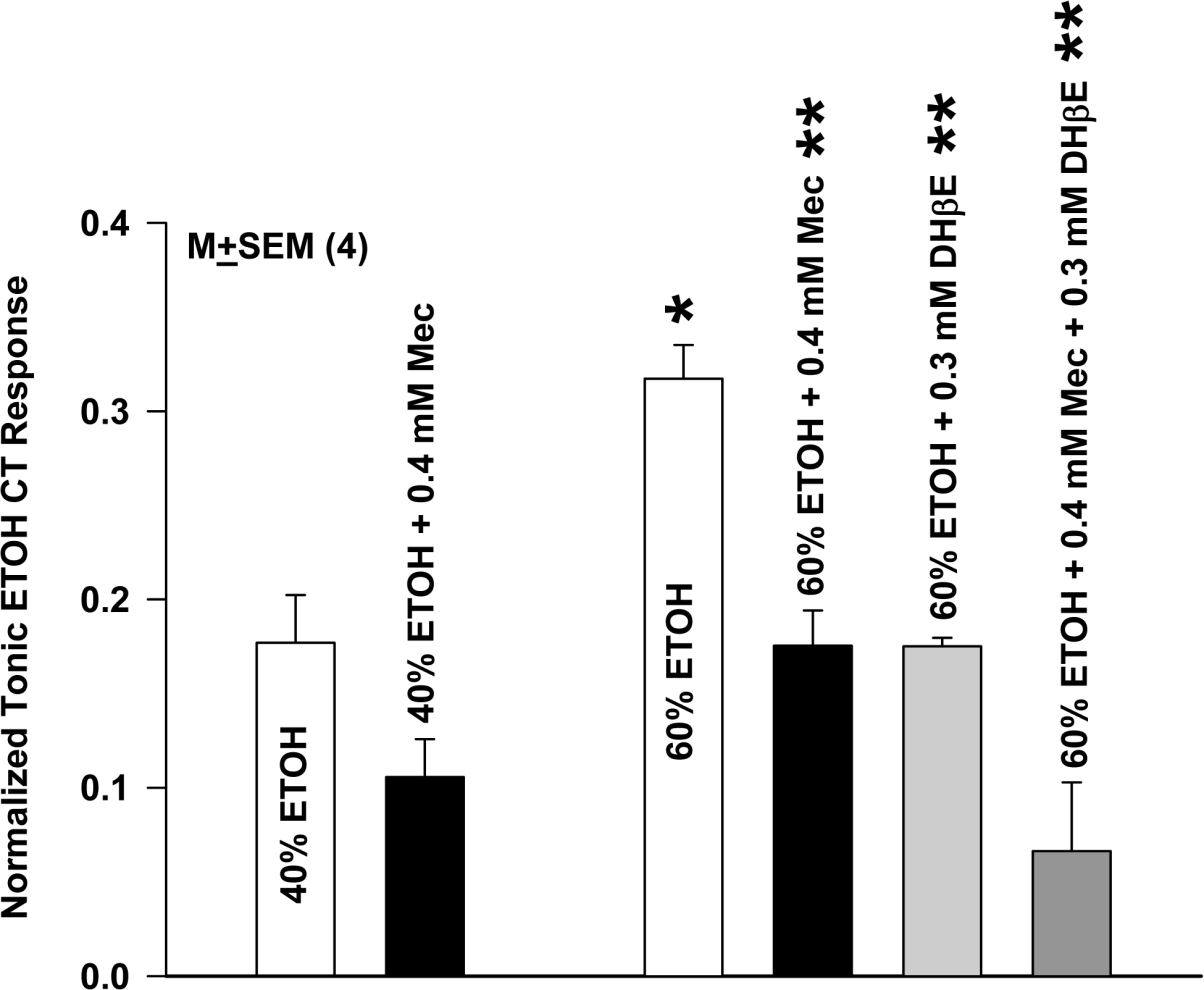
Effect of Mec and DHβE on the rat CT responses to ethanol (ETOH). The mean normalized CT responses (*N* = 4) at 40% and 60% ethanol in the presence and absence of 0.4 mM Mec and 0.3 mM DHβE. The mean tonic CT response at 60% ethanol was greater than the CT response at 40% ethanol (*p = 0.01; unpaired). Relative to 60% ETOH the **p values in the presence of 0.4 mM Mec, 0.3 mM DHβE, and 0.4 mM Mec + 0.3 mM DHβE were 0.0001, 0.0086, and 0.0002, respectively. Relative to 60% ETOH + 0.4 mM Mec and 60% ETOH + 0.3 mM DHβE, the p value at 60% ETOH + 0.4 mM Mec + 0.3 mM DHβE was 0.03 (unpaired; *N* = 4).

The nAChRs represent common targets at which acetylcholine, nicotine and ethanol functionally interact in the central nervous system [20]. Accordingly, we next tested if CT responses to ethanol are also sensitive to Mec and DHβE. Consistent with previous studies [30], stimulating the rat tongue with 60% ethanol (ETOH) produced a greater normalized tonic CT response than 40% ethanol (Fig 5). Adding 0.4 mM Mec inhibited the tonic CT response to 40% and 60% ethanol by 40.2% and 44.7%, respectively, relative to control. Adding 0.3 mM DHβE inhibited the CT response to 60% ethanol by 44.8%. Stimulating the tongue with 60% ethanol containing 0.4 mM Mec + 0.3 mM DHβE produced a further decrease in the ethanol CT response by 79.1% relative to control. These results indicate that similar to the CT responses to nicotine and acetylcholine, CT responses to ethanol are also inhibited by Mec and DHβE.

### CT responses to nicotine and ethanol are blocked by CP-601932

**Fig 6:**
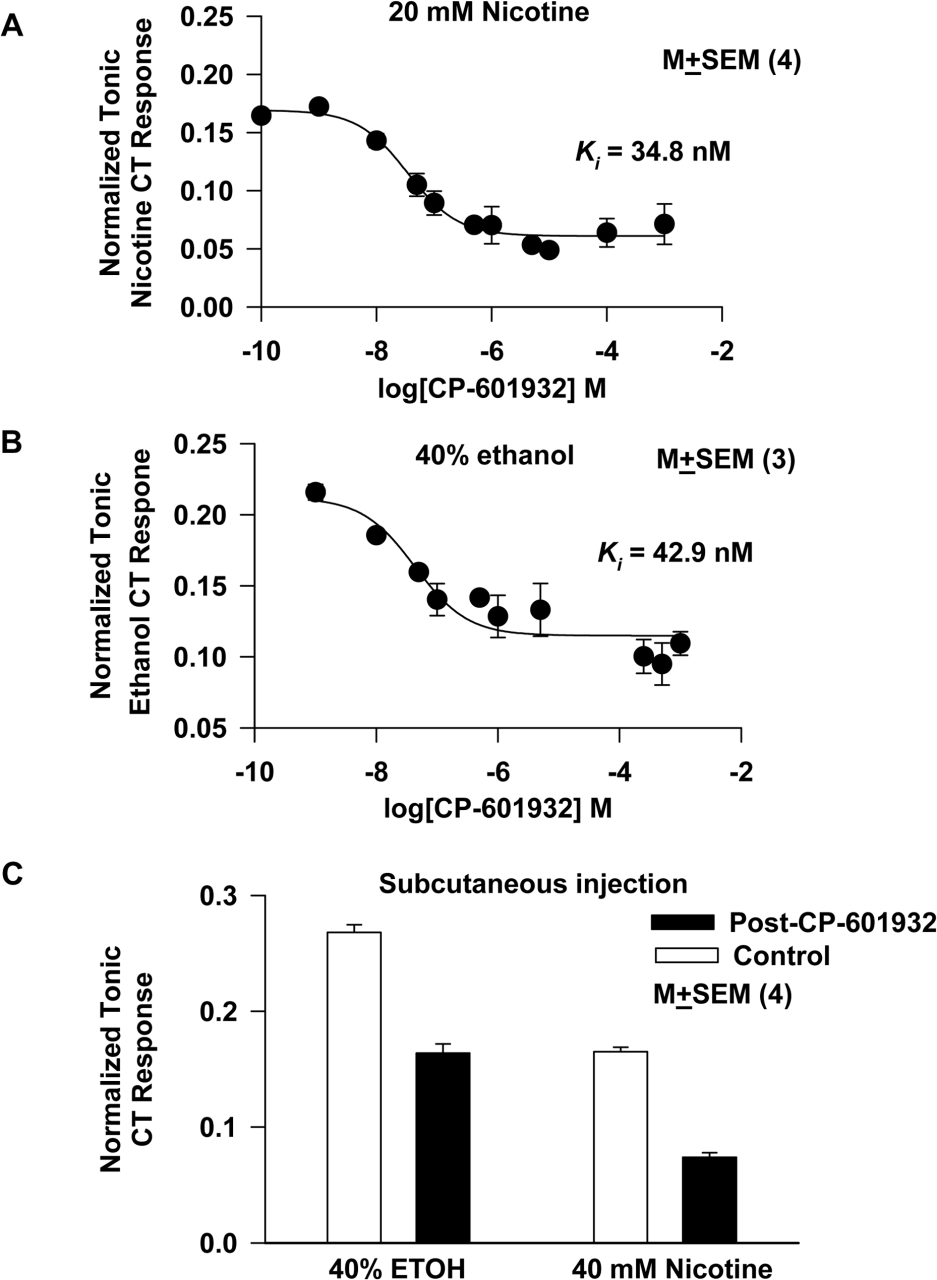
Effect of CP-601932 on the rat CT response to nicotine and ethanol. The mean normalized CT responses at 40 mM nicotine **(A)** or 40% ethanol **(B)** in the presence and absence of varying concentrations of CP-601932 (0–0.1 mM). CP-601932 decreased the normalized tonic component of the nicotine and ethanol CT response in a dose-dependent manner. Responses at each CP-601932 concentration were fitted to response inhibition curves (see text for details). For 40 mM nicotine the fit (R^2^ = 0.99) yielded an inhibitor-nAChR dissociation constant (*K*_*i*_) value of 34.8 nM and for 40% ethanol the fit (R^2^ = 0.88) gave a *K*_*i*_ value of 42.9 nM. These *K*_*i*_ values are within the range of values reported for *α*4*β*2 nAChRs [Chatterjee et al. 2011]. All mean normalized CT responses to 20 mM nicotine in the presence of CP-601932 at concentrations greater than 10^−8^ M were significantly smaller than the control values at p values <0.023. The p values for CP-601932 concentrations between 10^−6.3^ M and 10^−5^ M were 0.0001. The p values for CP-601932 concentrations at 10^−4^ M and 10^−3^ M were 0.0012 and 0.0055, respectively. For 40% ethanol, the p value for CP-601932 concentrations greater than 10^−8^ M was 0.0001 (unpaired). **(C)** Shows the mean normalized CT responses to 40% ethanol (ETOH) and 40 mM nicotine before and after 2h subcutaneous injection of saline (Control) or CP-601932 (10 mg/Kg body weight; Post-CP-601932). Relative to control the *p values for 40% ethanol and 40 mM nicotine were 0.0008 and 0.0005, respectively (unpaired; *N* = 4).

Rat CT responses to 20 mM nicotine or 40% ethanol were monitored in the presence of increasing concentrations (0–0.01 mM) of CP-601932, a partial agonist of the α3β4* nAChR. The mean normalized tonic CT responses to nicotine (Fig 6A) or ethanol (Fig 6B) were plotted as a function of the log[CP-601932] concentration in moles/liter. In each case the data were fitted to a response-inhibition curve (similar to Eq 2 but with *n* = 1 and with transformations to log (base 10) of the inhibitor concentration and the inhibitor-nAChR dissociation constant, *K*_*i*_ where the subscript i denotes an inhibition constant) [15]. CP-601932 inhibited the CT response to 20 mM nicotine and 40% ethanol in a dose-dependent manner with a *K*_*i*_ of 34.8 nM (Fig 6A) and 42.9 nM (Fig 6B), respectively (*N* = 3).

In a separate set of rats, CT responses to 40% ethanol or 40 mM nicotine were monitored before and after a 2 h subcutaneous injection of saline or CP-601932 (10 mg/Kg body weight) [7]. In CP-601932-treated rats, the CT responses to both ethanol and nicotine were inhibited by 38.9% and 55.2%, respectively, relative to saline injected rats (Fig 6C). Taken together, these results suggest that at these concentrations CP-601932, acting as an antagonist, inhibited rat CT responses to ethanol and nicotine when presented orally or injected subcutaneously.

### Regulation of nicotine and ethanol CT responses by intracellular cAMP and Ca^2+^

**Fig 7:**
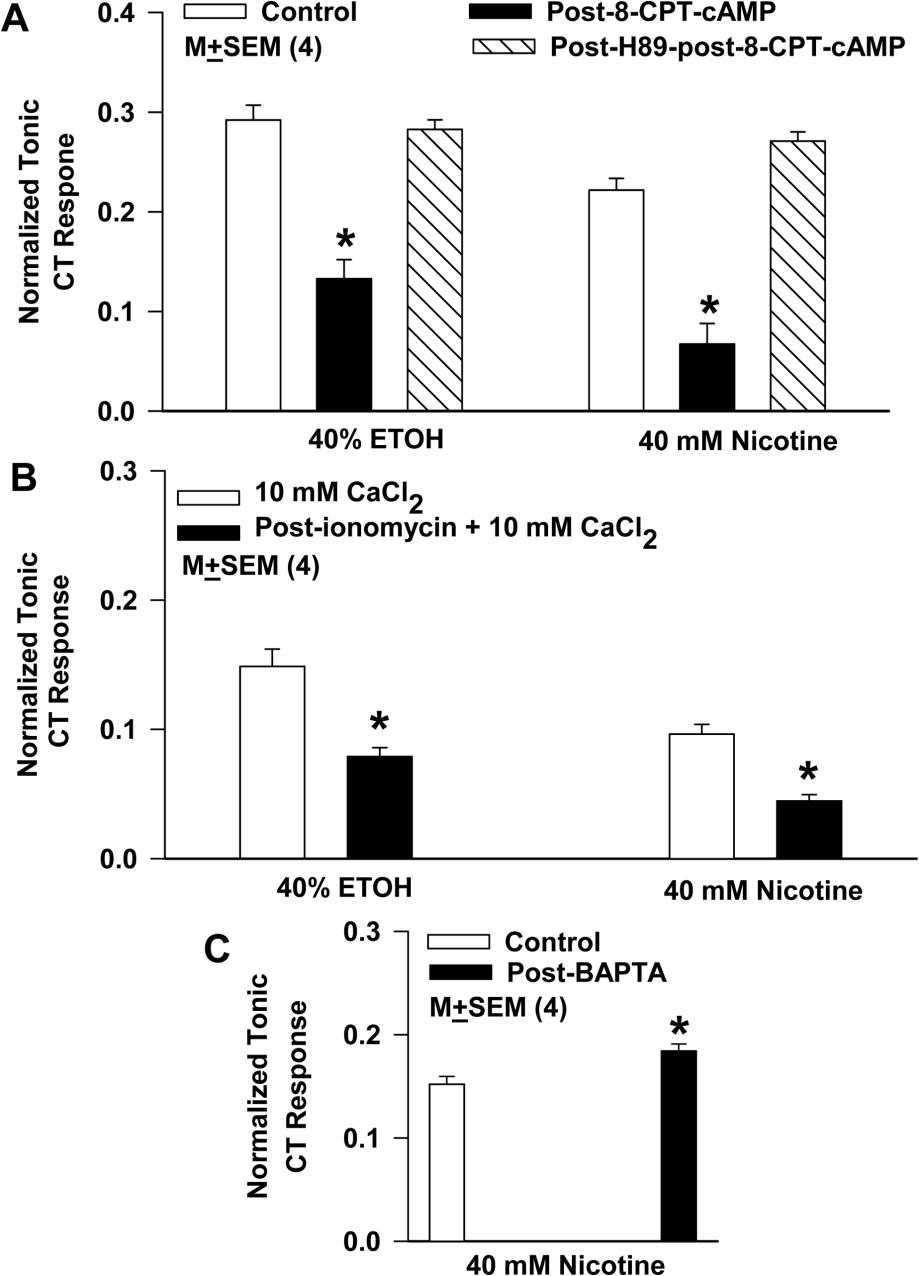
Effect of 8-CPT-cAMP, ionomycin + CaCl_2_, and BAPTA-AM on the rat CT response to ethanol (ETOH) and nicotine. **(A)** CT responses to 40% ethanol and 40 mM nicotine were monitored in rats before and after topical lingual application of 15 mM 8-CPT-cAMP for 30 min. The mean normalized tonic CT responses to ethanol and nicotine were inhibited relative to control (*p = 0.0001; *N* = 4). When rat tongues were pre-treated with 0.1 mM H89 before the topical lingual application of 8-CPT-cAMP, no significant changes in the CT response to ethanol and nicotine were observed relative to control. **(B)** CT responses to 40% ethanol + 10 mM CaCl_2_ and 40 mM nicotine + 10 mM CaCl_2_ were monitored in additional rats before and after topical lingual application of 0.15 mM ionomycin for 30 min. The mean normalized tonic CT responses to ethanol and nicotine were inhibited relative to control. Relative to control the *p values for ethanol and nicotine were 0.0035 and 0.0012, respectively (unpaired; *N* = 4). As in our earlier studies [4, 5], the addition of 10 mM CaCl_2_ to the nicotine and ethanol stimulation solutions did not alter the magnitude of the nicotine or ethanol CT response (data not shown). **(C)** CT responses 40 mM nicotine were monitored in additional group of rats before and after topical lingual application of 33 mM BAPTA for 30 min [4, 5]. BAPTA-AM treatment produced a small but significant (p = 0.019; unpaired; *N* = 4) increase in the normalized tonic CT response to nicotine relative to control.

We next tested if common intracellular signaling molecules, cAMP and Ca^2+^ that regulate CT responses to salty [22, 24], sour [22], and bitter [23] taste stimuli also regulate CT responses to alcohol and nicotine. CT responses in rats to 40 mM nicotine or 40% ethanol were monitored before and after topical lingual application of 15 mM 8-CPT-cAMP for 30 min. Following 8-CPT-cAMP treatment, there was a 54.5% decrease in the CT response to nicotine and a 69.7% decrease in ethanol CT response relative to control (Fig 7A; *N* = 4). Pretreating rat tongues with 0.1 mM H89 for 15 min before exposing them to 15 mM 8-CPT-cAMP for 30 min did not elicit the expected decrease in the CT response to nicotine or ethanol (Fig 7A; *N* = 4).

Rat CT responses to 40% ethanol and 40 mM nicotine containing 10 mM CaCl_2_ were monitored before and after topical lingual application of 150 μM ionomycin (Fig 7B). CT responses to both ethanol and nicotine were inhibited by 46.9 and 53.7% relative to control (Fig 7B; *N* = 4). As in our earlier studies [22, 23], the addition of 10 mM CaCl_2_ to the nicotine and ethanol stimulation solutions did not alter the magnitude of the nicotine or ethanol CT response (data not shown).

In rats topical lingual application of 33 mM BAPTA-AM for 30 min [22, 23] produced a small, but significant (p = 0.019; *N* = 4) enhancement in the CT response to 40 mM nicotine (Fig 7C). Taken together, these data indicate that CT responses to nicotine and ethanol are modulated by changes in TRC cAMP and Ca^2+^, presumably by changes in the phosphorylation state of the putative nAChRs via protein kinase A (PKA) and/or protein kinase C (PKC) dependent mechanisms [31, 32].

### Patch-clamp studies on isolated rat fungiform TRCs

Whole nerve recordings do not provide information about responses in individual TRCs. Therefore, the patch clamp method [27, 28] was used to investigate the effect of nicotine in isolated fungiform TRCs. We recorded from 10 individual TRCs in physiological solutions, and applied voltage steps between -50 mV and +50 mV from a holding potential of -80 mV. Eight TRCs did not elicit alterations in current at the holding potential or in response to depolarizing steps when exposed to nicotine between 0.05 and 0.5 mM in the Tyrode solution (S1A and S1B Fig) Two taste cells responded with an increase in inward current when exposed to nicotine. In a representative trace (S1D Fig), nicotine (0.2 mM) elicited inward currents that were inhibited in the presence of 0.3 mM Mec (S1E Fig). S1F Fig shows the current (I) versus voltage (V) plot under control conditions (0 Nic), in the presence of 0.2 mM Nic and in the presence of 0.2 mM Nic + 0.3 mM Mec. The nicotine-induced current reversed near -10 mV, as expected for poorly selective nAChR channels. Thus, nicotine elicits currents in a subset of fungiform taste bud cells by activating nAChRs. These currents are most likely related to the CT responses to nicotine and their sensitivity to Mec.

## Discussion

The data presented here indicate that CT responses to nicotine, ethanol and acetylcholine depend in part on the presence of nAChRs expressed in a subset of fungiform TRCs. These results further support the conclusion that in addition to the classical T2R-GPCR-dependent bitter taste transduction pathway, there is a parallel TRPM5-independent but nAChR-dependent bitter taste transduction pathway (Figs 1–6) that is important for detecting the bitter taste of nicotine [33, 34] and ethanol [35, 36].

In contrast to the data shown here with nicotine (Figs 1 and 2), while WT mice responded with a concentration-dependent increase in the tonic CT response to lingual stimulation with 10 mM and 20 mM quinine, no significant increase in the tonic CT response was observed above the rinse baseline in TRPM5 KO mice. In addition, Mec had no effect on the CT responses to 10 mM and 20 mM quinine in WT mice [15]. In behavioral studies, 10 mM quinine was aversive to WT mice but not for TRPM5 KO mice. Nicotine was aversive for both genotypes when tested against water and only in KO mice when tested against quinine. In contrast, WT mice preferred nicotine over quinine. Thus, nicotine was equally aversive for KO and WT mice, showing that behavioral responses to nicotine are modulated by a TRPM5-independent mechanism [15].

In contrast to nicotine, the quinine CT response depends entirely on the T2R-TRPM5 bitter taste transduction pathway. There is no contribution from the TRPM5-independent or the nAChR-dependent bitter taste pathways to the quinine CT response [15, 21]. Consistent with this, quinine CT responses in rats could be inhibited by triphenylphosphine oxide (TPPO), a specific blocker of TRPM5 [37] and by chelating TRC Ca^2+^ by BAPTA-AM [23]. These results show that even in rats, the quinine CT response depends entirely on the T2R-TRPM5 bitter taste transduction pathway.

As in our previous studies [15], the current studies were performed using TRPM5 KO mice lacking exons 15–19 encoding transmembrane segments 1–5 [21]. In a previous study, both TRPM5 KO mice and the PLCβ2 KO mice lacked behavioral and peripheral neural responses to quinine and other prototypical bitter tastants [21]. In contrast, the TRPM5 KO mice null for TRPM5 protein expression due to deletion of TRPM5's promoter region and exons 1–4 (including the translation start site) were indifferent to all concentrations of quinine up to the maximum solubility of 10 mM in initial licking tests [38]. In addition, the whole CT nerve responses to quinine hydrochloride and denatonium benzoate at 0.1, 1, 10 and 20 mM were not different between WT and TRPM5 KO mice. However, TRPM5 KO mice demonstrated greatly diminished glossopharyngeal nerve responses to quinine hydrochloride and denatonium benzonate relative to WT mice. The TRPM5 KO mice showed no glossopharyngeal nerve responses to 1 mM quinine hydrochloride and minimal responses to 10 mM quinine hydrochloride and to 10 and 20 mM denatonium benzoate [38]. In additional studies, using the above TRPM5 KO mice construct [39, 40] CT responses to quinine in TRPM5 KO mice were diminished but were not abolished. Taken together, the studies in the above TRPM5 KO mice construct tend to suggest that while in the posterior circumvallate field, glossopharyngeal nerve responses to quinine depend upon both TRPM5-dependent and TRPM5-independent pathways, in the anterior fungiform field quinine responses are independent of TRPM5. It is suggested that in TRPM5 KO mice lacking exons 15–19 encoding transmembrane segments 1–5, in which the expressed truncated amino terminal portion of TRPM5 inhibits the activity of other TRP channels expressed in TRCs. Another potential explanation for differences between the two constructs of TRPM5 KO mice may stem from the fact that the mice used in this and other studies [15, 21] are apparently a mixture of C57BL/6 and the unspecified strain of ES cells, while the mice used in the study by Damak et al [38] are 100% C57BL/6.

Our data demonstrate that in WT mice, CT responses to quinine are not affected by Mec, indicating that the taste responses to quinine are independent of nAChRs expressed in fungiform TRCs [15]. Also, in behavioral studies, both WT and TRPM5 KO mice were able to distinguish between nicotine and quinine [15]. Further studies are needed in the TRPM5 KO mice null for TRPM5 protein expression due to deletion of TRPM5's promoter region and exons 1–4 (including the translation start site) to determine if nicotine responses in the absence or presence of nAChR antagonists are same or different between the two constructs of TRPM5 KO mice.

Our results indicate that CT responses in WT mice and SD rats are derived from both the T2R-TRPM5-dependent pathway and the T2R-TRPM5-independent pathway, with the latter pathway depending on the expression of nAChRs in a subset of fungiform taste bud cells. Although the mean tonic CT response to nicotine was of similar magnitude in SD rats and WT mice, the contribution of T2R-bitter taste transduction mechanism to the tonic CT response in rats was much smaller than in WT mice. Our results further suggest that the contribution of the T2R-dependent and the nAChR-dependent pathways to the CT response to nicotine varies in SD rats and WT mice and within the two genotypes varies with the nicotine concentration because of the different kinetics connected with each receptor type (Fig 1 and Table 1). In a recent study [41] nicotine was shown to inhibit TRPM5 currents with an effective inhibitory concentration of ~1.3 mM at −50 mV. It is suggested that this effect may contribute to the inhibitory effect of nicotine on gustatory responses in therapeutic and experimental settings, where nicotine is often employed at millimolar concentrations. Consistent with our results, the above observations of Gees et al [41] also imply the existence of a TRPM5-independent pathway for the detection of nicotine bitterness.

Since nicotine [42] and ethanol [43] can easily permeate cell membranes and cross cells, they could possibly act on TRCs or at the CT nerve terminals surrounding the taste buds. Acetylcholine, presumably acting in its well-established role as a parasympathetic nervous system neurotransmitter, has been proposed to modulate TRCs via muscarinic AChRs [44]. Our studies suggest that acetylcholine, acting as an apically applied taste stimulus, also elicits CT responses that depend on nAChRs. Acetylcholine, a quaternary ammonium cation ester, is expected to have a significantly lower membrane permeability compared to nicotine, and in the absence of a specific transporter for acetylcholine, is not expected to rapidly permeate apical taste cell membranes. Thus, it is unlikely that acetylcholine, presented apically, can directly activate nAChRs in the CT taste nerve. Our patch-clamp data (S1 Fig) demonstrate that nAChRs, expressed specifically in TRCs, can serve as receptors for nicotine and work as neuronal nAChRs that form ligand-gated ion channels in the plasma membranes of these TRCs. Being ionotropic receptors, nAChRs are effectively ligand-gated ion channels that do not make use of a second messenger as metabotropic receptors do. It is important to note that ethanol, nicotine and acetylcholine elicit CT responses when dissolved in deionized water. This suggests that CT responses to ethanol, nicotine and acetylcholine do not depend upon the presence of cations in the apical compartment. However, nAChRs localized in the basolateral membrane could function as ligand-gated ion channels resulting in Na^+^, K^+^ or Ca^2+^ entry across the basolateral membrane of TRCs. Such a mechanism would require taste ligands to enter TRCs or at least to cross tight junctions, at present an area of uncertainty.

Our data using the nAChR modulators: Mec, DHβE, and CP-601932 (Figs 1–6) suggest that multiple nAChR subunits contribute to the alcohol and nicotine CT response. In our earlier studies, Mec did not alter CT responses to NaCl, quinine and SC45647 (an artificial sweetener) [15]. These results indicate that the effects of nAChR antagonism are quite specific for nicotine. The effects of Mec, DHβE, and CP-601932 were reversible, suggesting that these effects are not due to prolonged nAChR desensitization.

Neuronal nAChRs can be homopentamers or heteropentamers and function as fast ionotropic cationic nicotinic receptor channels. To date, eight α-like subunits termed α2, α3, α4, α5, α6, α7, α9, and α10 (α8, an avian nAChR has not been found in mammals) and three non-α subunits termed β2, β3, and β4 have been cloned from neuronal tissues [12, 20]. Currently, the identity of the functional nAChRs in TRCs is lacking. However, our data suggest that DHβE-sensitive nAChRs containing α4β2 and CP-601932-sensitive nAChRs containing α3β4* subunits are most likely involved in the nicotine- and ethanol-induced increase in the CT response resulting from membrane depolarization on activation of nAChRs [45].

Both partial agonists of α3β4*-type nAChRs, CP-601932 and PF-4575180 selectively decreased ethanol but not sucrose consumption and operant self-administration following long-term exposure [7]. In Wistar rats, trained to self-administer 10% ethanol, a subcutaneous injection of CP-601932 (10 mg/kg) was administered 30 min before the start of the operant session. CP-601932 significantly decreased the number of presses on the active lever and inhibited 10% ethanol self-administration relative to saline injected rats. In our studies, using the above experimental protocol, CP-601932 inhibited the CT responses to both nicotine and ethanol (Fig 6C). These results suggest that part of the change in behavioral response to ethanol reported by Chatterjee et al. [7] may depend on CP-601932-induced changes in α3β4* nAChRs in TRCs.

An increase in TRC cAMP inhibited the CT response to nicotine and ethanol. This inhibition was not observed if the tongue was pretreated with the PKA inhibitor H89 (Fig 7A). We have previously shown that topical lingual application of 8-CPT-cAMP or forskolin + 3-isobutyl-1-methylxanthine (IBMX) specifically enhanced the amiloride- and benzamil-sensitive NaCl CT responses without altering the benzamil-insensitive NaCl CT response [24]. Topical lingual application of 8-CPT-cAMP also enhanced the CT response to strong acids (HCl) without altering the responses to weak organic acid (acetic acid and CO_2_) [46]. These data suggest that an increase in TRC cAMP has a differential effect on neural responses to stimuli representing different taste qualities.

The nAChRs can be phosphorylated by PKA, PKC, and protein tyrosine kinases [31, 32]. The nAChR purified from rat brains was shown to be phosphorylated *in vitro* by cAMP-dependent PKA. PKA specifically phosphorylated nAChRs on the α4 subunits, and H8, an inhibitor of PKA, inhibited completely the phosphorylation [47]. Depending on the nAChR type, cAMP can either potentiate or attenuate the nAChRs. Alpha 7 nicotinic receptors were potentiated both by cAMP and PKC [48]. In rat pinealocytes, acetylcholine or the selective nicotinic receptor agonist 1,1-dimethyl-4-phenylpiperazinium iodide (DMPP) activated large nAChR currents in whole cell patch-clamp experiments. Norepinephrine reduced the nAChR currents, an effect partially mimicked by a β-adrenergic receptor agonist, isoproterenol. Increasing intracellular cAMP levels using membrane-permeable 8-bromoadenosine (8-Br)-cAMP or 5,6-dichlorobenzimidazole riboside-3',5'-cyclic monophosphorothioate (cBIMPS) reduced nAChR activity. PKA inhibitors, H-89 and Rp-cAMPS, blocked the modulation of nAChR by adrenergic stimulation [49]. Stimulation of PKC may lead to speeding of nAChRs desensitization [31] and a slowing of desensitization with chronic increase of cAMP [50].

Loading TRCs with Ca^2+^ using ionomycin inhibited CT responses to both nicotine and ethanol (Fig 7B). In contrast, decreasing TRC [Ca^2+^]_i_ by BAPTA-AM produced a small but significant increase in the CT response to nicotine (Fig 7C). We have previously shown that the ionomycin-induced increase in TRC Ca^2+^ inhibits the benzamil-sensitive NaCl CT response [22] and the tonic CT response to sour taste stimuli (HCl, acetic acid and CO_2_) [22]. In contrast, the ionomycin-induced increase in TRC Ca^2+^ had no effect on the CT responses to sucrose, quinine and denatonium [23]. Decreasing TRC Ca^2+^ with BAPTA-AM treatment enhanced the magnitude of the benzamil-sensitive NaCl CT response [22] and inhibited the tonic CT response to acidic stimuli [22] and quinine [23]. With respect to the benzamil-insensitive NaCl CT response, an increase in TRC Ca^2+^ inhibited and a decrease in TRC Ca^2+^ enhanced the resiniferatoxin-induced increase in the NaCl CT response [51]. These data suggest that changes in cytosolic Ca^2+^ in TRCs have a differential effect on neural responses to stimuli representing different taste qualities. Changes in cytosolic Ca^2+^ can either directly modulate the ion channel, as in the case of the benzamil-sensitive epithelial Na^+^ channel [52], or via a change in the phosphorylation state of the ion channel through modulation of a kinase or a phosphatase activity, as in the case of the resiniferatoxin effects on the Bz-insensitive NaCl CT response [51].

It is important to note that while loading TRCs with BAPTA-AM inhibited the tonic CT response to quinine [23], it enhanced the CT response to nicotine (Fig 7C). This further suggests that the bitter tastants quinine and nicotine have different transduction mechanisms [15].

It is suggested that Ca^2+^ entry through both nAChRs and voltage-gated Ca^2+^ channels exerts a negative feedback on nAChR activity through stabilization of desensitized states [53]. Alternately, Ca^2+^ effects may be induced by secondary activation of one or more Ca^2+^-dependent kinases or phosphatases [31, 32]. Further studies are needed to delineate these mechanisms in TRCs. It is suggested that CT-mediated taste input may not be necessary for long term maintenance of ethanol self-administration [54]. However, the CT data presented here may be of relevance to the initiation of ethanol drinking and very first experiences with nicotine from cigarette smoking, chewing tobacco or from other sources. Thus, altering the taste of nicotine by modulating nAChRs in the anterior or posterior taste field may delay or prevent initiation of ethanol drinking or use of nicotine containing products.

We have previously [15] shown that quinine was not aversive to TRPM5 KO mice. However, nicotine was equally aversive to WT and TRPM5 KO mice. Although CT responses to nicotine were reduced in TRPM5 KO mice, they were not abolished unlike responses to quinine, which were absent. Furthermore, CT responses to nicotine were inhibited by the nAChR-antagonist, Mec, in WT mice, TRPM5 KO mice and in rats. The effects of Mec were also found to be behaviorally relevant. In both WT and TRPM5 KO mice Mec significantly reduced the aversive effects of nicotine [15]. In human subjects, Mec was reported to increase nicotine preference and to attenuate nicotine discrimination [55]. We have previously shown that alcohol preferring (P) rats elicit greater CT responses to ethanol and show preference for 5% ethanol relative to alcohol nonpreferring (NP) rats [56]. These differences between P and NP rats may be partly due to differences in the expression levels of nAChRs in taste bud cells [57].

Besides the TRPM5-dependent and TRPM5-independent bitter taste transduction pathways described above, nicotine activates capsaicin-sensitive trigeminal neurons [58, 59] and TRPA1 [60]. In the presence of the general nAChR blocker hexamethonium, nociceptive neurons showed nicotine-induced responses that were strongly reduced in TRPA1-deficient mice [60]. In our previous study [15], the contribution of somatosensory input was investigated by measuring behavioral responses to nicotine in adult WT and TRPM5 KO animals that had been injected with capsaicin as neonates. This treatment produces systemic and life-long elimination of the majority of capsaicin-sensitive neurons, causing deficits in chemonociceptive reactivity [61]. Although responses to capsaicin solutions confirmed the treatment was effective, preference for 0.5 and 1 mM nicotine did not differ between untreated and capsaicin-treated TRPM5 KO animals. Given that TRPM5 KO animals, irrespective of a reduction in capsaicin-sensitive somatosensory neurons, retained an aversion to nicotine, it suggests that at these concentrations, alternate TRPM5-independent sensory pathways, presumably taste-related, participate in the detection of nicotine [15].

In summary, we provide evidence that suggests that nAChRs represent common molecular targets in TRCs and are involved in detecting the bitter taste of nicotine, ethanol and acetylcholine. We hypothesize that this nAChR-dependent, but TRPM5-independent bitter taste transduction pathway may also play a role in nicotine and ethanol addiction, comorbidity and relapse. Further neural and behavioral studies are needed in nAChR KO mice to directly demonstrate that these pathways are TRPM5-independent.

## Supporting Information

S1 Fig Effect of nicotine on isolated single fungiform TRCs. Isolated TRCs were bathed in Tyrode solution and the pipette solution contained (in mM): 140 KCl, 11 EGTA, 10 HEPES, 5 MgATP, 2 MgCl_2_, 1 CaCl_2_ (pH 7.2). The holding potential was -80 mV and voltage steps were applied between -50 mV and +50 mV. We recorded from 10 individual TRCs. Eight TRCs did not elicit any currents when exposed to nicotine (Nic) between 0.05 and 0.5 mM in the Tyrode solution. A representative trace of a non-responding TRC is shown at 0 **(A)** and 0.2 mM **(B)** nicotine. Two cells responded with an increase in inward currents when exposed to nicotine. In a representative trace **(C, D and E)**, 0.2 mM nicotine (Nic) elicited currents in an isolated fungiform TRC that were inhibited in the presence of 0.3 mM Mec. **(F)** Shows the I/V relationships under control condition (0 Nic), in the presence of 0.2 mM nicotine, and in the presence of 0.2 mM nicotine + 0.3 mM Mec. Note that *in vitro* isolated TRCs respond to nicotine in the micromolar range and in the millimolar range in the CT experiments *in vivo*.
(TIF)
