# Supplementary figures and images for: Nicotinic Acetylcholine Receptor (nAChR) Dependent Chorda Tympani Taste Nerve Responses to Nicotine, Ethanol and Acetylcholine
Source: PLoS One. 2015 Jun 3;10(6):e0127936. doi: 10.1371/journal.pone.0127936 (PMC4454666; doi:10.1371/journal.pone.0127936)

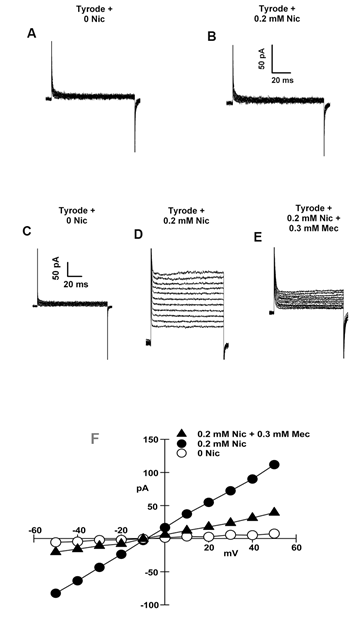

Supplement: S1 Fig — Isolated TRCs were bathed in Tyrode solution and the pipette solution contained (in mM): 140 KCl, 11 EGTA, 10 HEPES, 5 MgATP, 2 MgCl2, 1 CaCl2 (pH 7.2). The holding potential was -80 mV and voltage steps were applied between -50 mV and +50 mV. We recorded from 10 individual TRCs. Eight TRCs did not elicit any currents when exposed to nicotine (Nic) between 0.05 and 0.5 mM in the Tyrode solution. A representative trace of a non-responding TRC is shown at 0 (A) and 0.2 mM (B) nicotine. Two cells responded with an increase in inward currents when exposed to nicotine. In a representative trace (C, D and E), 0.2 mM nicotine (Nic) elicited currents in an isolated fungiform TRC that were inhibited in the presence of 0.3 mM Mec. (F) Shows the I/V relationships under control condition (0 Nic), in the presence of 0.2 mM nicotine, and in the presence of 0.2 mM nicotine + 0.3 mM Mec. Note that in vitro isolated TRCs respond to nicotine in the micromolar range and in the millimolar range in the CT experiments in vivo. (TIF) [file pone.0127936.s001.tif]
